# Graphene Plasmonics in Sensor Applications: A Review

**DOI:** 10.3390/s20123563

**Published:** 2020-06-23

**Authors:** Shinpei Ogawa, Shoichiro Fukushima, Masaaki Shimatani

**Affiliations:** Advanced Technology R&D Center, Mitsubishi Electric Corporation, 8-1-1 Tsukaguchi-Honmachi, Amagasaki, Hyogo 661-8661, Japan; Fukushima.Shoichiro@cb.MitsubishiElectric.co.jp (S.F.); Shimatani.Masaaki@bk.MitsubishiElectric.co.jp (M.S.)

**Keywords:** graphene, plasmonics, photoelectric sensors, biological sensors, 2D materials

## Abstract

Surface plasmon polaritons (SPPs) can be generated in graphene at frequencies in the mid-infrared to terahertz range, which is not possible using conventional plasmonic materials such as noble metals. Moreover, the lifetime and confinement volume of such SPPs are much longer and smaller, respectively, than those in metals. For these reasons, graphene plasmonics has potential applications in novel plasmonic sensors and various concepts have been proposed. This review paper examines the potential of such graphene plasmonics with regard to the development of novel high-performance sensors. The theoretical background is summarized and the intrinsic nature of graphene plasmons, interactions between graphene and SPPs induced by metallic nanostructures and the electrical control of SPPs by adjusting the Fermi level of graphene are discussed. Subsequently, the development of optical sensors, biological sensors and important components such as absorbers/emitters and reconfigurable optical mirrors for use in new sensor systems are reviewed. Finally, future challenges related to the fabrication of graphene-based devices as well as various advanced optical devices incorporating other two-dimensional materials are examined. This review is intended to assist researchers in both industry and academia in the design and development of novel sensors based on graphene plasmonics.

## 1. Introduction

Plasmonics is an important technology that permits the manipulation of photons beyond the diffraction limit [1,2,3]. Surface plasmon polaritons (SPPs) and surface plasmon resonance (SPR) are associated with the interactions of electromagnetic waves with the interface between a plasmonic material and a dielectric as a result of the coupling of photons and electrons. Various uses have been proposed for these phenomena in a wide range of applications, including in the fields of biology [4] and chemistry [5] and in gas sensing [6], image sensing [7,8] and optical communication devices [9]. Conventional plasmonic materials such as Au, Ag and Al can produce SPPs at wavelengths lower than the mid-infrared (IR) region of the spectrum. However, there is a significant demand for SPPs in the mid-IR and terahertz (THz) regions [10,11,12]. Moreover, the electrical tuning of SPP wavelengths would revolutionize present-day plasmonic devices [13]. Graphene-based plasmonics has thus received increasing attention in both industry and academia as these challenges are addressed.

**Figure 1:**
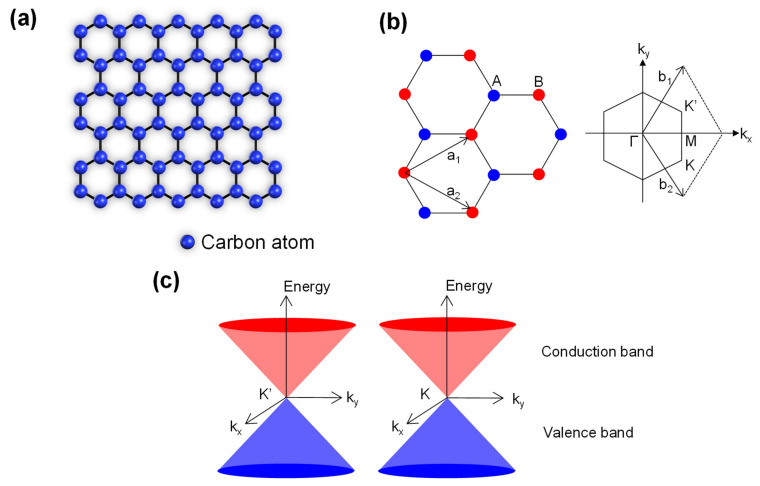
(**a**) A schematic illustration of the atomic arrangement in graphene. (**b**) The honeycomb lattice (left) and the Brillouin zone (right) for graphene. a_1_ and a_2_ are the lattice unit vectors. b_1_ and b_2_ are the reciprocal lattice vectors. (**c**) The energy bands in the vicinity of the K and K’ points.

As shown in Figure 1a–c, graphene comprises atomically-thin carbon sheets having a hexagonal lattice structure. This material exhibits unique optical and electrical properties because of its Dirac cone-type bandgap structure, where the energy depends linearly on the wave vector around the K point [14,15,16]. Specifically, graphene has exceptional electrical, optical and chemical properties that are well-suited to sensor applications and that are not achievable with conventional technologies based on bulk materials [17]. These characteristics include high carrier mobility, fast photoresponse, broadband photodetection from the ultraviolet to the THz regions of the spectrum, flexibility and low cost [18]. Thus, graphene and other two-dimensional materials are the focus of an increasing number of research studies and novel phenomena associated with these materials are still being discovered [19]. At the same time, new applications such as high-speed transistors [20], photodetectors [21,22], biological sensors [23,24], gas sensors [25], energy storage [26], displays [27] and flexible devices [28] are being proposed based on the novel physics of such materials [29,30].

In particular, graphene can serve as a plasmonic material operating in the mid-IR to THz regions of the spectrum and its optical constants (such as permittivity and refractive index) can be adjusted by applying a gate voltage [31], which cannot be achieved by conventional metal-based plasmonic materials. Therefore, graphene-based plasmonics-type sensors show promise with regard to meeting the recent demand for devices operating in the mid-IR to THz regions with electrically tunable wavelengths.

Recent advances in graphene plasmonics can be roughly classified into three areas, which involve the application of—(i) intrinsic plasmons in graphene (meaning that the graphene itself functions as a plasmonic material), (ii) the interactions between graphene and SPPs induced by metallic nanostructures and (iii) the electrical control of SPPs induced by metallic nanostructures. Each of these fields of study can be applied to the design of specific sensors and, in the case of (ii) and (iii), various metamaterials [32,33] or metasurfaces [34,35] are integrated with graphene. The purpose of this review paper is to examine the progress to date in graphene-based plasmonics-type sensors, as well as to summarize the theoretical background according to the above three principles, focusing primarily on optical and biological sensors. Graphene-based reconfigurable devices such as absorbers and reflectors are also introduced because these devices are important to future sensing systems. A number of excellent review papers [31,36,37,38] and books [39,40] on this topic have already been published but the majority are centered on the fundamental physics of graphene SPPs. In contrast, the present paper assesses the applications of graphene in this regard and focuses on the development of novel sensors and the expansion of graphene plasmonics applications. Thus, the advantages and future challenges associated with graphene plasmonics in sensor applications are also addressed herein.

## 2. Theoretical Background

This section briefly discusses the theoretical background of graphene SPPs so as to clarify the advantages of this phenomenon in graphene and the aspects in which it differs from that in conventional noble metals. 

### 2.1. Intrinsic SPPs in Graphene

It is helpful to begin by examining conventional SPPs, because intrinsic graphene SPPs can be best understood by comparison to SPPs induced in noble metals. The bulk plasma frequency, ω_p_, of metals can be written as:

(1) $$\omega_{p}=\sqrt{\frac{Ne^{2}}{\epsilon_{0}m_{e}^{*}}},$$

where *N*, *e*, *ε*_0_ and m_e_* are the free carrier concentration, the electron charge, the permittivity of free space and the electron effective mass, respectively. The SPP frequency is $\omega_{sp}=\frac{\omega_{p}}{\sqrt{2}}$ and the permittivity of the metal, $\epsilon_{m}=1-\frac{\omega_{p}^{2}}{\omega^{2}},$ is obtained from the Drude model. Generally, the real and imaginary parts of the permittivity of a plasmonic material must be negative and small, respectively. If these conditions are satisfied, the SPP mode at the interface between a dielectric and a plasmonic material can be obtained from Maxwell’s equations. The permittivity of the plasmonic material determined in this manner will establish the optical response of the material [41]. The plasmon dispersion relationship can also be obtained from Maxwell’s equations in conjunction with specific boundary conditions and is written as:

(2) $$q=\frac{\omega }{c}\;\sqrt{\frac{\epsilon_{d}\epsilon_{m}}{\epsilon_{d}+\epsilon_{m}}},$$

where *q*, *ε_d_* and *ε_m_* are the SPP wave vector and the permittivity values of the background dielectric and the metal, respectively. 

At this point, we can examine intrinsic graphene SPPs. The optical conductivity of graphene, *σ_g_*, can be described as in Equations (3)–(7), which are derived from the Kubo formula [39,42,43,44]. This conductivity has both intraband (*σ_intra_*) and interband (*σ_inter_*) transitions, such that:

(3) $$\sigma_{g}=\sigma_{\mathrm{intra}}+\sigma_{\mathrm{inter}},$$

where

(4) σintra=σ0π4ℏΥ−iℏωEF+2kBTln1+e−EFkBT

and

(5) $$\sigma_{\mathrm{inter}}=\sigma_{0}\left[G\left(\hbar \omega /2\right)+i\frac{4\hbar \omega }{\pi }\int_{0}^{\infty }dE\frac{G\left(E\right)-G\left(\hbar \omega /2\right)}{{\left(\hbar \omega \right)}^{2}-4E^{2}}\right].$$

Here,

(6) $$G\left(x\right)=\frac{\sinh\left(\frac{x}{k_{B}T}\right)}{cosh\left(\frac{E_{F}}{k_{B}T}\right)+cosh\left(\frac{x}{k_{B}T}\right)},$$

(7) $$\sigma_{0}=\frac{e^{2}}{4\hbar },$$

and *Υ*_,_ *ℏ*, *k_B_*, *T* and *E_F_* are the relaxation rate, the reduced Plank constant, the Boltzmann constant, temperature and the Fermi energy, respectively.

In case of $2E_{F}>\hbar \omega$, the contribution of interband transitions can be negligible due to Pauli blocking. Working in the mid-IR and THz regions at room temperature and with typical doping levels, the optical conductivity of graphene can be obtained using the Drude model [39] as:

(8) $$\sigma_{g}=\sigma_{0}\frac{4i}{\pi }\frac{E_{F}}{\hbar \omega +i\hbar \Upsilon }.$$

**Figure 2:**
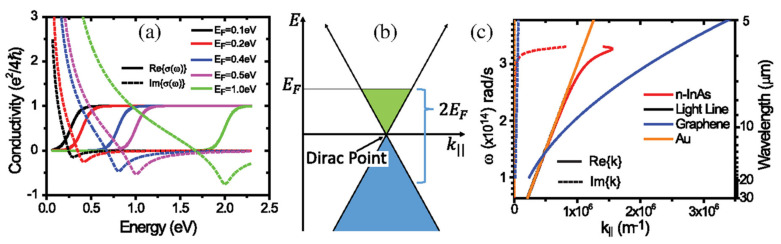
(**a**) The real and imaginary parts of the optical conductivity of graphene for various values of *E_F_*. (**b**) The energy band structure of graphene, showing the Dirac point and Fermi level. (**c**) A comparison of the plasmon dispersion results for graphene and for various other materials. (**a**,**b**) are adapted from Reference [41]. © 2020 Society of Photo-Optical Instrument Engineers (SPIE).

Figure 2a,b plot the optical conductivity calculated for graphene for various Fermi levels, *E_F_* [41] and the energy band structure of graphene, respectively. Here, $E_{F}=\hbar \nu_{F}k_{F}$, where $\nu_{F}$ is the Fermi velocity of approximately 10^6^ m/s and $k_{F\;}=\sqrt{\pi n}$ is the Fermi wave vector (where *n* is the free carrier concentration). At higher energy ($\hbar \omega >2E_{F}$), the real part of the optical conductivity maintains a constant value and is attributed to interband transitions [45].

If it is assumed that the graphene is sandwiched between sheets of an asymmetrical dielectric material (with dielectric constants $\epsilon_{1}$ and $\epsilon_{2}$), the dielectric constant can be replaced by their average value, $\epsilon =\left(\epsilon_{1}+\epsilon_{2}\right)/2$. The graphene plasmon dispersion relationship in the case that $q\gg \;\frac{\sqrt{\in }\omega }{c}$ can be described using the same method as applied with metals [46], as:

(9) $$q=\frac{2\pi \hbar^{2}\epsilon_{0}\epsilon }{e^{2}E_{F}}\;\left(1+\frac{i\Upsilon }{\omega }\right)\omega^{2}.$$

Figure 2c plots the plasmon dispersion data calculated for graphene surrounded by air and for n-doped InAs and Au [41].

SPPs in conventional plasmonic metals such as Ag and Au and Al occur at visible and near-IR, and ultraviolet wavelengths, respectively [38]. Figure 2c indicates that the graphene SPPs occur in the mid-IR wavelength range and exhibit significant electromagnetic wave confinement. A number of interesting studies have examined the limits of graphene SPPs [47,48,49]. According to reference [49], the wavelength of a graphene SPP will be 66 times smaller than the illumination wavelength, while the lifetime of a graphene SPP is 1600 fs at 60 K and the propagation length of an intrinsic plasmonic is over 10 μm. In contrast, in the SPP wavelength range, the confinement factor and the lifetime for Ag SPPs are ~1 and 14 fs, respectively, at 10 K [49]. These superior properties of graphene SPPs suggest applications in a variety of sensors [50]. It is worth noting that the coupling of graphene SPPs with incident light from free space is difficult. Near-field scanning microscopy [47,48], graphene nanoribbons (GNRs) [51], triangular wedges [52] and graphene nano disks [53] have all been used in attempts to address this issue.

Equation (9) can be used to express the bulk plasma frequency based on the Drude model if the loss of graphene is ignored (γ=0). Consequently, the plasma frequency of graphene can be described as [38,39]:

(10) $$\omega_{p}=\sqrt{\frac{e^{2}E_{F}q}{2\epsilon \epsilon_{0}\pi \hbar^{2}}}.$$

This equation demonstrates why graphene and metals have different *ω_p_* values. Specifically, *ω_p_* depends on *q*, is proportional to N14 (because EF∝N12) and is independent of the effective mass of the electron. However, this equation does contain Plank’s constant, which is attributed to the Dirac nature of graphene SPPs [38]. 

### 2.2. Interactions of Graphene and Plasmonic Structures

**Figure 3:**
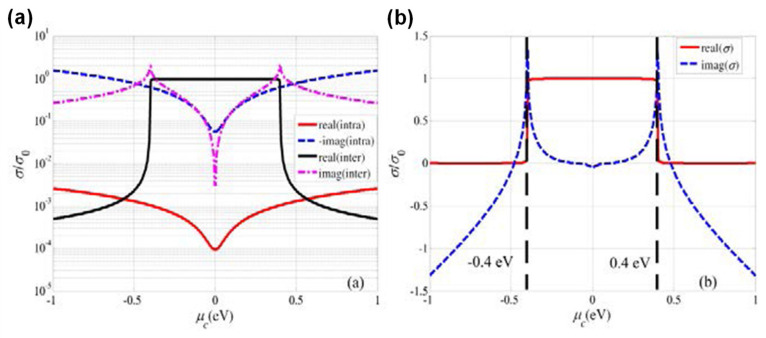
Calculated optical conductivity values for graphene at a wavelength of 1.55 μm and a temperature of 300 K as functions of chemical potential. (**a**) The real and imaginary parts of the interband and intraband transition contributions. (**b**) The overall optical conductivity values. Both figures were adapted with permission from Reference [54]. © 2020 Optical Society of America.

The *E_F_* value of graphene can be tuned by electrostatic gating. As a result, the optical constant of graphene can also be adjusted. In addition, SPPs are sensitive to changes in the refractive index of the surrounding materials and so the interaction between the structure into which the graphene is integrated and the SPPs is an important aspect of sensor applications. In particular, this phenomenon may permit the development of electrically-tunable graphene-based plasmonics-type sensors. Figure 3a,b plot calculated values of optical conductivity for graphene based on Equations (3)–(5) as functions of the chemical potential, *μ_c_*, of graphene at a temperature of 300 K and a wavelength of 1.55 μm [54]. Note that these optical conductivity values have been normalized relative to *σ_0_*. The chemical potential of graphene which can be adjusted by electrostatic gating and is calculated as [55]:

(11) $$\left|\mu_{c}\right|≅\;\left|E_{F}\right|=\hbar \nu_{F}\sqrt{\pi \left|a_{0}\left(V_{g}-V_{\mathrm{Dirac}}\right)\right|},$$

where *V_g_* and *V_Dirac_* are the applied voltage and the voltage at the Dirac point, respectively, and *a_0_* is a constant with a value of approximately 9 × 10^−16^ m^−2^V^−1^.

As shown in Figure 3, the optical conductivity of graphene can be tuned by varying the applied voltage. This effect allows modification of graphene SPPs as well as SPPs induced by metals and also enables electrical control of the reflection, transmission, absorption and phase of an SPP-based sensor. The sensor applications associated with such tuning are reviewed in Section 5.2 and Section 6.

## 3. Optical Sensors

One of the drawbacks of graphene is its low absorbance of approximately 2.3% and this low responsivity must be addressed to allow graphene to be used in optical sensors. Various approaches to mitigating this problem have been proposed, including thermoelectric systems using hetero-electrodes [56,57], bolometric effects [58], PN junctions [59,60], integration with waveguides [61,62,63], photogating [64,65,66,67,68,69], heterojunctions [70,71] and SPPs. Of particular interest are two methods that take advantage of SPPs based on concepts (i) and (ii) as introduced in Section 1 and discussed in Section 3.1 and Section 3.2, respectively. In general, graphene-based optical sensors are based on field effect transistors (FETs) with graphene channels formed on SiO_2_/Si substrates. Applying a voltage between source and drain electrodes and a back gate results in changes in the channel that are correlated with incident light, while the back gate voltage controls the Fermi level of the graphene.

### 3.1. Graphene-Based Optical Sensors

**Figure 4:**
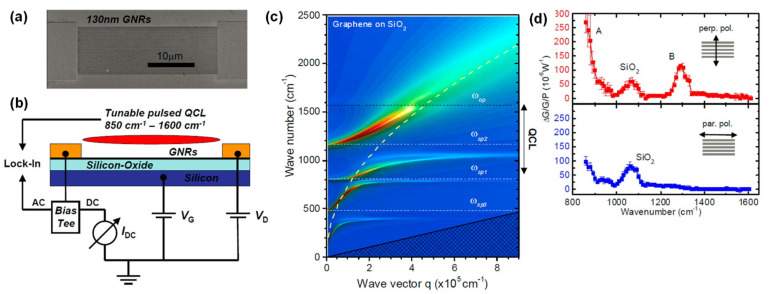
(**a**) A scanning electron microscopy (SEM) image of a graphene nanoribbon (GNR) channel and electrode. (**b**) A schematic of the concept for a GNR-based plasmonic IR detector. (**c**) Dispersion relation for hybrid plasmon-phonon modes in graphene on SiO_2_. (**d**) The polarization dependence of the normalized photocurrent. All figures are adapted from reference [72]. © 2020 American Chemical Society.

Incident light cannot directly couple with graphene SPPs and so GNRs or micropatches are commonly used for the excitation of graphene SPPs, which can enhance the responsivity of GNRs-based photodetectors. Figure 4a,b present a scanning electron microscopy (SEM) image of a GNR channel with an electrode and a schematic illustration of a GNR-based mid-IR photodetector, respectively [72]. To produce this detector, a GNR was formed on a SiO_2_ layer on a Si substrate. SiO_2_ generates phonon polaritons that can couple with graphene SPPs, as shown in Figure 4c. The dashed yellow curve corresponds to graphene plasmon dispersion without hybridization of the SiO_2_ phonons. *ω_op_* is the graphene optical phonon energy. The sp^0^, sp^1^ and sp^2^ phonons are polar SiO_2_ phonons that interact with graphene plasmons. Anti-crossing behavior clearly appears in the SiO_2_ phonon modes, which is attributed to phono-plasmon interactions [73,74]. This phonon-plasmon coupling is vital to the functioning of FET-type graphene-based devices. A temperature increase is induced by this coupling and is four times larger than that for conventional graphene IR detectors. Enhancement of the output photocurrent as a function of the gate voltage and graphene Fermi level for various nanoribbon widths has been reported [73]. The results indicated that hybrid plasmon–phonon modes are readily tunable by adjusting the gate voltage. Consequently, both higher responsivity and room-temperature operation can be successfully achieved using GNR SPPs in conjunction with the phonon modes of a polar substrate. In addition, GNR SPPs exhibit a polarization dependence. As shown in Figure 4d, the photocurrent can be enhanced using only the electric–field component perpendicular to the GNR axis. As a result, it is possible to detect only s-polarized light. Typically, this polarization dependence would be expected to degrade the performance of an optical sensor intended to detect non-polarized light. However, if this polarization effect can be properly employed, it may be possible to realize polarimetric image sensors capable of distinguishing artificial materials from natural environments or recognizing human faces [75,76,77].

GNRs-based photodetector can also operate in THz region [78]. GNRs covered with HfO_2_, which has a high k value, have also demonstrated room temperature operation and broadband detection from the visible to mid-IR regions [79]. This performance is attributed to the persistent high mobility in the graphene as a result of the reduced carrier scattering in GNRs capped by HfO_2_. Moreover, GNRs combined with nanodisks have demonstrated higher performance at room temperature in the case that mid-IR SPPs are employed [80]. It should be noted that the heat generated by SPPs or phonons in the mid-IR wavelength region plays an important role in the room-temperature performance of a sensor. In fact, this effect is vital to achieving high-performance IR sensors operating under ambient conditions.

### 3.2. Graphene Integrated with Plasmonic Structure-Based Optical Sensors

**Figure 5:**
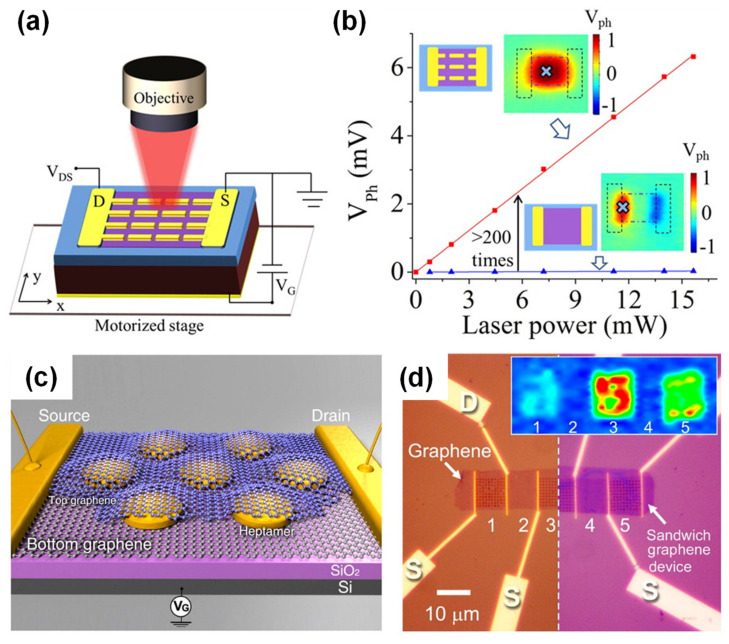
Graphene integrated metasurface photodetectors. (**a**) A schematic of a periodic micropatch array and (**b**) the performance data for field effect transistor (FET)-based mid-IR photodetectors with periodic micropatches. (**c**) A schematic illustration and (**d**) optical microscopy image of a graphene-based optical sensor made from gold heptamers sandwiched between two graphene monolayers. (**a**,**b**) are adapted from reference [85]. © 2020 American Chemical Society. (**c**,**d**) are adapted from Reference [86]. © 2020 American Chemical Society.

The integration of graphene with plasmonic structures such as periodic metal patches and metal-insulator-metal (MIM) units has been widely investigated as a means of enhancing the responsivity of graphene-based photodetectors or realizing wavelength- or polarization-selective functions [7,81,82]. Various plasmonics structures have been examined, including one-dimensional metal stripes [83,84], two-dimensional (2D) metal micropatches [85], heptamers [86], metal fractal patterns [87], bull’s eyes [88], Si quantum dots [89] and MIM [90,91] designs. However, the underlying principle for all such techniques is the same, in that graphene absorption is enhanced due to the significant confinement of the electromagnetic resonance of the graphene by localized SPR in the vicinity of such plasmonics structures. This increased absorbance mitigates the inherent low responsivity of graphene-based optical sensors, based on principle (ii) as described in the Introduction. Figure 5a,b present a schematic illustration of a graphene-based optical sensor with 2D periodic Au micropatches and a comparison of the output voltages obtained with and without these plasmonic patterns, respectively. The data indicate that the responsivity in the mid-IR wavelength region is increased by a factor of more than 200. Figure 5c,d show a schematic illustration and optical microscopy image of a graphene-based optical sensor made from gold heptamers sandwiched between two monolayers of graphene, respectively. This structure increased the graphene responsivity by 800% at visible wavelengths. It should also be noted that the enhancement factor greatly depends on the source-drain and back gate voltages that are applied, as well as the quality of the graphene, meaning that it is difficult to directly compare responsivity values between different papers. However, it is at least evident that SPR induced by metal nanostructures can enhance the responsivity of graphene-based optical sensors.

In addition to enhancement of the absorption of graphene by localized SPR, the thermoelectric effect induced by the metal nanostructures may contribute to the high responsivity and room temperature operation because of strong localization of the electromagnetic field in SPR [92].

Another approach to improving the responsivity of graphene based on the use of plasmonic structures is to integrate plasmonic waveguides [93,94] or nanogap structures into the sensor [95]. Plasmonic structures with narrow gaps induce gap resonance modes that can enhance absorption. At present, the majority of graphene-based optical sensors are single-pixel devices. However, in the near future, advanced image sensors [96] using the techniques described above are expected to become commercially available despite some remaining challenges [97,98].

## 4. Biological Sensors

**Figure 6:**
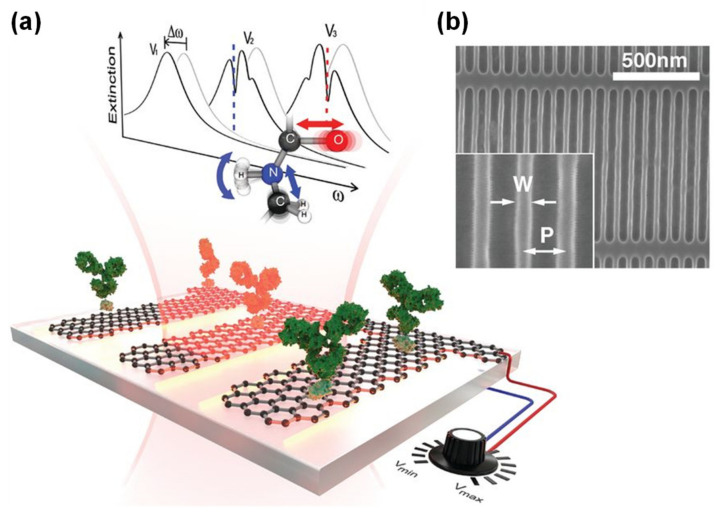
(**a**) A schematic showing the concept of a GNR–based biosensor and (**b**) an SEM image of a GNR array. Both figures are adapted from Reference [100]. © 2020 The American Association for the Advancement of Science.

Graphene has also shown promise as a component of biological sensors, as it has a large surface area and is biocompatible with antibodies, enzymes, DNA, cells and proteins [24]. In particular, surface-enhanced infrared absorption (SEIRA) can significantly enhance the sensitivity in the IR wavelength range using SPPs [99]. Therefore, graphene plasmonics has become an important component of the recent development of biological sensors. Figure 6 [100] presents a schematic of a proposed GNR-based biosensor that takes advantage of graphene SPPs [100,101]. Incident IR irradiation generates SPR across the GNRs. The electromagnetic field is strongly localized at the GNR edges, which enhances the interaction between the IR rays and the protein molecules adsorbed on the GNRs. Such sensors are based on principles (i) and (iii) as outlined in the Introduction. In these devices, the electromagnetic field generated by the graphene SPPs is concentrated at the GNR edges and increases the light-matter interactions of molecules adsorbed on the graphene. Proteins can be recognized by monitoring the SPR spectral shifts associated with narrow dips corresponding to the molecular vibration bands of the protein. Moreover, the gate voltage tunability of graphene-based SPR enables continuous sweeping over the protein vibrational bands [100].

Other well-known types of graphene-based biological sensors comprise graphene integrated with metasurfaces [102,103] and plasmonic metal films [104,105]. The former devices are based on principles (ii) and (iii) outlined in the Introduction. One well-known application is surface enhanced Raman spectroscopy (SERS) [106]. Graphene-based substrates with plasmonic nanostructures are promising owing to their high sensitivity, stability, reproducibility and fluorescence quenching [107]. The integration of graphene with metasurfaces can provide more design freedom and the interactions between graphene and SPPs induced by other metallic nanostructures are reviewed in Section 5. The latter sensors take advantage of graphene as a passivation and functionalization film in order to enhance the quality of the sensing surface. In this type of device, graphene SPPs do not appear but rather the sensitivity of metallic SPR is increased.

As discussed in this section, biological sensors using graphene SPPs operate on the same principles as other optical sensors or devices. Therefore, present-day optical units based on graphene SPPs have significant potential in drug discovery, food safety analysis and the monitoring of bio-reactions.

## 5. Absorbers and Emitters

Graphene-based absorbers are very similar to graphene-based photoelectric sensors in terms of the enhancement of the absorbers. Graphene-based emitters are also important light sources in various analytical applications, such as gas analysis systems. Both graphene-based emitters and absorbers operate on the same principle based on Plank’s law [108] and the present section focuses on absorbers. The majority of graphene-based absorbers incorporate plasmonic metamaterials or metasurface absorbers (PMAs) that operate according to principles (ii) and (iii) as explained in the Introduction. These devices can be classified into two groups according to their function—(1) those that increase absorption by enhancing the quantum efficiency of graphene and (2) reconfigurable optical devices involving the modulation of the absorption wavelength.

### 5.1. Absorption Enhancement

**Figure 7:**
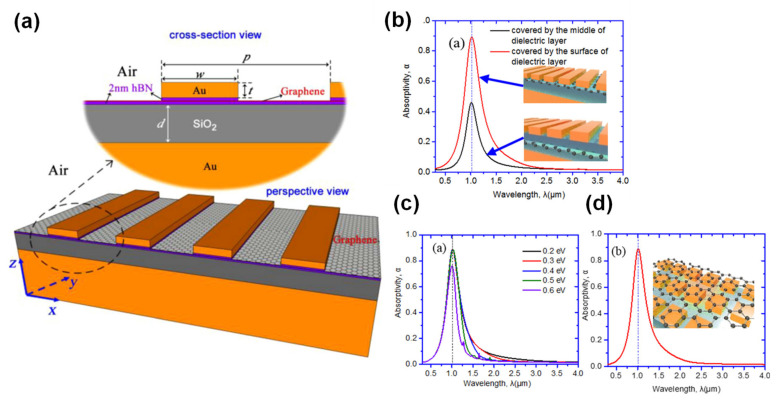
Graphene integrated metamaterial absorbers. (**a**) A schematic of graphene embedded in an MIM absorber using hexagonal boron nitride (hBN) and (**b**) schematics of graphene coated over top of MIM absorbers together with absorbance data for graphene in the middle and the top of the insulator layers. (**c**) The absorbance plots of this device for various chemical potentials. (**d**) The absorbance of graphene coated over top of metal-insulator-metal (MIM) absorbers. (**a**) is reprinted from Reference [115] with the permission of AIP Publishing. (**b**–**d**) were adapted with permission from Reference [118]. © 2020 Optical Society of America.

PMAs have attracted significant interest because they exhibit near-unity absorbance along with wavelength selectivity while allowing small device dimensions [99,109,110]. Although graphene-based PMAs without metal-based plasmonic structures have been investigated, various types of PMAs with graphene have also been researched. These have included one-dimensional gratings [111], 2D plasmonic crystals [112,113], bow-tie antennae and MIM structures [114,115,116,117,118]. These devices are being examined because graphene-based PMAs without metal-based plasmonic structures have narrow operation wavelength bands [53] and low absorbance values [119]. The majority of such units use MIM-based PMAs and Figure 7 presents a typical example of a MIM-based PMA incorporating graphene. Figure 7a,b,d provide schematic illustrations of the graphene layers formed on the top of the device [115], on the top and in the middle of the insulator layers and on the top of the micropatches [118].

Interestingly, the absorbance of such devices will change according to the location of the graphene layers, indicating that the localized SPPs (LSSPs) induced by the MIM-based PMA can enhance the absorption of the graphene. It is important to note that the positioning of the graphene and the thickness of the insulator layer are also important factors, in addition to the chemical potential of the graphene resulting from matching of the surface optical impedance [120]. These devices can function both as high-performance electromagnetic absorbers and as advanced thermal IR or THz sensors. Figure 7c plots the absorbance data for various graphene chemical potentials and demonstrates the possibility of electrically tuning the absorbance, as discussed in the next section.

### 5.2. Absorption Wavelength Modulation

**Figure 8:**
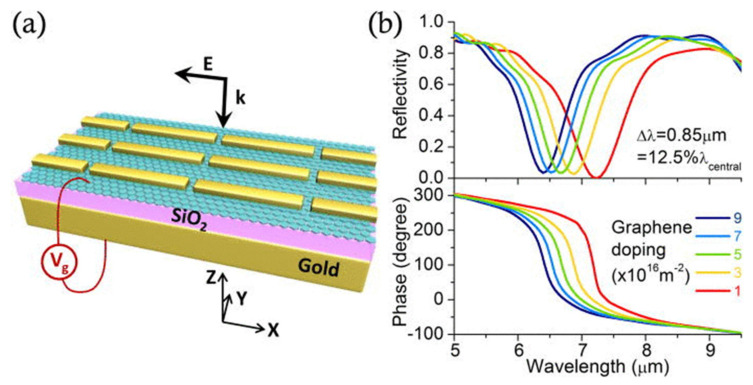
(**a**) A schematic of a reflect-array of rod antennas integrated with graphene. (**b**) The calculated reflection spectra (upper panel) and phase of the reflected light (lower panel) for various graphene doping levels. Both figures are reprinted from Reference [123] with the permission of AIP Publishing.

As discussed in Section 2.2, the optical conductivity of graphene can be tuned by applying a voltage, such that the modulation of SPP wavelengths can be achieved using metallic nanostructures. Typically, this modulation is associated with the integration of graphene with metasurfaces [120,121,122,123,124,125,126,127], split rings [122,128,129] or other metamaterials [130,131,132,133,134] or with metallic nanoparticles [135,136]. Figure 8 shows a typical example of the modulation of the device reflection and the reflection phase by adjusting the extent of doping of the graphene. The device structure consists of a MIM-based PMA with embedded graphene, which is similar to those in Figure 7.

Figure 8b demonstrates that both the reflection and the phase can be significantly adjusted by modifying the extent of doping. Interestingly, this same effect can be realized using metasurfaces without gold plates as substrates. Although the surface conductivity of graphene is minimal, the interaction is strengthened when graphene interacts with the strong electric fields of SPPs generated by currents in the metallic nanostructures [122]. This electrical tuning effect can be used in a wide range of applications, such as advanced IR, biological [137] and gas sensors, as well as in other optical devices including band stop filters [138], waveguides [139,140] and cloaking units [141]. In particular, the electrical control of the phase of the reflected light could potentially permit dynamic control of the light direction in future sensing systems, as discussed in the next section.

## 6. Reconfigurable Reflectors

There is presently a high demand for electrically-tunable optical devices, especially for reconfigurable mirrors suitable for solid-state light detection and ranging (LIDAR) applications [142]. Consequently, various metasurfaces have been proposed for the arbitral control of reflected or transmitted light [143,144,145]. These metasurfaces when integrated with graphene could allow the electrical tuning of light direction using principles (ii) and (iii), as discussed in the Introduction [146,147,148,149]. In this regard, graphene also offers an additional advantage in that it does not affect the optical constant of the overall structure but rather modulates only the resonance because of the extreme thinness of graphene layers.

**Figure 9:**
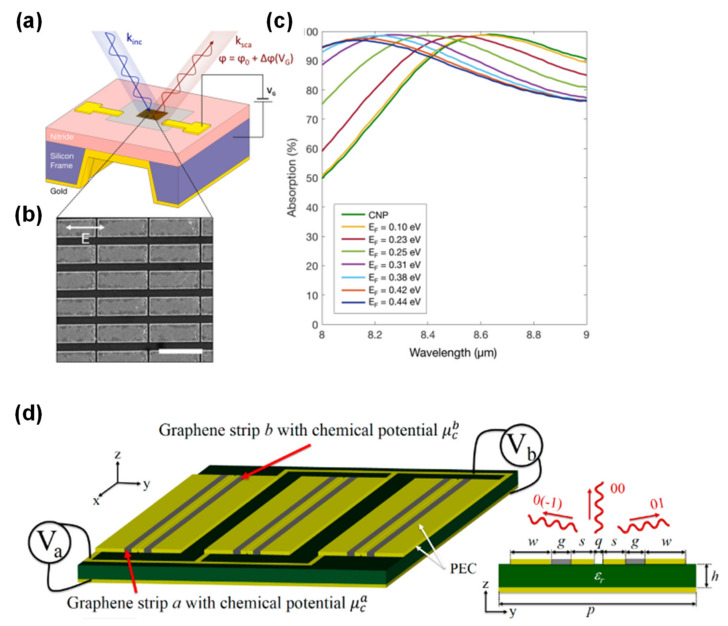
(**a**) A schematic illustration of a phase modulator based on graphene embedded with metallic resonators. (**b**) An SEM image of the resonators. (**c**) The absorption spectra for various graphene Fermi levels. (**d**) A schematic illustration of a reconfigurable reflector based on graphene embedded in MIM-based metasurfaces. (**a**–**c**) are adapted from Reference [147]. © 2020 American Chemical Society. (**d**) is adapted from reference [148]. © 2020 American Chemical Society.

The majority of metasurfaces used for the purpose of beam steering are based on the concept of gradients, meaning that specific surface patterns are topologically engineered using the general version of Snell’s law [143]. Figure 9a–c show a gate-tunable graphene-gold resonator [147]. The gold resonator array is formed on graphene channel on SiNx. Applying a voltage to the graphene in this device changes the graphene’s chemical potential, which in turn modulates the phase of the SPR induced by the metallic nanostructure. A 237° phase modulation range was demonstrated at an operating wavelength of 8.50 μm using this concept, while an array comprising a set of these resonators realized a 30° steering angle. Figure 9d presents images for electrically reconfigurable metagratings using graphene and metallic stripes operating in the THz range [148]. The device has a structure similar to that for a MIM-based PMA with graphene but the graphene acts as an electrical connection between the metal stripes. In such a device, incident light can be rerouted in the desired direction with over 80% efficiency, including the losses associated with the graphene.

This same concept can be employed in other wavelength regions, such as the visible and near-IR. However, there are still challenges associated with this technology, including achieving high efficiencies, the suppression of higher order diffractions, variations in the uniformity of the graphene and the appropriate fabrication of the metallic nanostructures. Control of the transmission of light [150] or light focusing [151], such as normally provided by a lens, could also be realized based on this concept. The resulting beam steering devices in conjunction with optical sensors could be used in advanced driver assistance systems and drone technology.

## 7. Future Outlook

Graphene SPPs are significantly affected by the quality of the graphene [152,153]. Therefore, the fabrication of both hexagonal boron nitride (hBN) as well as graphene will be very important with regard to the development of graphene-based plasmonic devices in the future because hBN has an atomically smooth surface and a similar lattice constant to that of graphene, making it the most suitable substrate for graphene. Fortunately, graphene growth technology has greatly matured in recent years [154,155,156]. Moreover, turbostratic graphene has been proposed as a means of suppressing deleterious electrical disturbances in oxide layers under graphene [157,158]. However, there are still a number of important challenges that must be addressed in order to integrate silicon-based semiconductor technology with graphene in terms of uniformity and size [159]. Although high-quality hBN can currently be obtained only by exfoliation [160], recent advances in chemical vapor deposition techniques for the synthesis of hBN [161] represent a key technology for the construction of graphene-based plasmonics-type devices. The intrinsic losses for graphene SPPs are also important issues for future graphene SPP-based sensors. Graphene encapsulated with hBN layers is effective for achieving high confinement and low SPP damping [152].

The combination of 2D materials such as hBN [153], transition metal dichalcogenides [162,163] and black phosphorous [164] with various polaritons, including SPPs, exciton polaritons and phonon polaritons, is also expected to allow the development of more advanced graphene-based plasmonic devices [162,165]. Heterostructures involving such materials together with bilayer graphene are also promising, because these have the potential to exhibit novel physical properties, such as valleytronics [166,167,168], magic angle effects [169], twisted graphene plasmons [170,171] and Moiré superlattices [172,173]. Devices based on direct electrical current control of graphene SPPs are promising for mid-IR and THz light sources for sensor systems [174,175,176]. Finally, advanced metasurfaces such as all-dielectric metasurfaces [145] are an important aspect of addressing the intrinsic losses associated with graphene SPPs. In addition, the inverse design method will assist in realizing advanced sensors or 2D heterostructures with high efficiencies that cannot be developed using conventional intuition-based approaches [177,178].

## 8. Conclusions

This review examined graphene plasmonics with regard to potential sensors applications. This phenomenon has opened up new fields concerning the use of SPPs in the mid-IR and THz regions, as a direct result of the Dirac cone band structure of graphene. As a consequence, high-performance optical sensors operating at IR wavelengths and at ambient temperature can be realized using graphene SPPs induced by GNRs. Graphene-based optical sensors with plasmonic nanostructures also show promise as high-responsivity wavelength- or polarization selective functional devices. Biological sensors based on graphene SPPs also exhibit higher performance and wavelength-tunable operation. In addition, work to date shows that graphene-based absorbers and reconfigurable absorbers will be important components of future sensing systems with applications in gas analysis, chemical analysis, alcohol detection and LiDAR. Taking advantage of graphene enables these optical components to operate at specific desired wavelengths as a result of electrostatic gating. Recent advances in other 2D materials as well as in the fabrication of heterostructures and in all-dielectric metasurfaces are expected to address the intrinsic loss effects associated with graphene SPPs and enable the development of next generation sensors having a wide range of uses, such as for the analysis of images, biological compounds, gases and chemicals,. It is hoped that this review will contribute to the development of advanced sensors using graphene plasmonics and the expansion of the range of associated applications.
